# Comparison of the Development of SARS-Coronavirus-2-Specific Cellular Immunity, and Central Memory CD4+ T-Cell Responses Following Infection versus Vaccination

**DOI:** 10.3390/vaccines9121439

**Published:** 2021-12-07

**Authors:** Kevin M. Dennehy, Eva Löll, Christine Dhillon, Johanna-Maria Classen, Tobias D. Warm, Lukas Schuierer, Alexander Hyhlik-Dürr, Christoph Römmele, Yvonne Gosslau, Elisabeth Kling, Reinhard Hoffmann

**Affiliations:** 1 Institute for Laboratory Medicine and Microbiology, Medical Faculty, University Hospital Augsburg, 86156 Augsburg, Germany; eva.loell@uk-augsburg.de (E.L.); lukas.schuierer@uk-augsburg.de (L.S.); elisabeth.kling@uk-augsburg.de (E.K.); reinhard.hoffmann@uk-augsburg.de (R.H.); 2 Department of Pathology, Medical Faculty, University Hospital Augsburg, 86156 Augsburg, Germany; christine.dhillon@uk-augsburg.de; 3 Internal Medicine III-Gastroenterology and Infectious Diseases, Medical Faculty, University Hospital Augsburg, 86156 Augsburg, Germany; Johanna-maria.classen@uk-augsburg.de (J.-M.C.); christoph.roemmele@uk-augsburg.de (C.R.); 4 Clinic for Vascular Surgery, Medical Faculty, University Hospital Augsburg, 86156 Augsburg, Germany; tobias.warm@uk-augsburg.de (T.D.W.); alexander.hyhlik-duerr@uk-augsburg.de (A.H.-D.); yvonne.gosslau@uk-augsburg.de (Y.G.)

**Keywords:** SARS-CoV-2, COVID-19, vaccination, cellular immunity, memory T-cells

## Abstract

Memory T-cell responses following infection with coronaviruses are reportedly long-lived and provide long-term protection against severe disease. Whether vaccination induces similar long-lived responses is not yet clear since, to date, there are limited data comparing memory CD4+ T-cell responses induced after SARS-CoV-2 infection versus following vaccination with BioNTech/Pfizer BNT162b2. We compared T-cell immune responses over time after infection or vaccination using ELISpot, and memory CD4+ T-cell responses three months after infection/vaccination using activation-induced marker flow cytometric assays. Levels of cytokine-producing T-cells were remarkably stable between three and twelve months after infection, and were comparable to IFNγ+ and IFNγ+IL-2+ T-cell responses but lower than IL-2+ T-cell responses at three months after vaccination. Consistent with this finding, vaccination and infection elicited comparable levels of SARS-CoV-2 specific CD4+ T-cells after three months in addition to comparable proportions of specific central memory CD4+ T-cells. By contrast, the proportions of specific effector memory CD4+ T-cells were significantly lower, whereas specific effector CD4+ T-cells were higher after infection than after vaccination. Our results suggest that T-cell responses—as measured by cytokine expression—and the frequencies of SARS-CoV-2-specific central memory CD4+T-cells—indicative of the formation of the long-lived memory T-cell compartment—are comparably induced after infection and vaccination.

## 1. Introduction

Throughout 2021, we had the unusual opportunity to concurrently measure the development of immune responses to SARS-coronavirus-2 (SARS-CoV-2) in patients after natural infection, as well as responses from hospital staff members after vaccination with BioNTech/Pfizer BNT162b2. The landmark papers describing the BNT162b2 vaccine compared SARS-CoV-2-specific antibody responses following infection and vaccination [1,2]. However, such studies did not compare cellular immune responses following vaccination and infection. More recent studies have compared the development of memory CD8+ T-cell responses after infection and vaccination, memory CD4+ T-cell responses of naïve and convalescent patients following vaccination, and, lastly, T-cell transcriptomic profiles and cytokine-producing T-cells following infection and vaccination [3,4,5,6]. As such, there are only few studies to date that have directly compared memory CD4+ T-cell responses, and in particular memory T-cell subsets following infection and vaccination.

Despite marked decreases in SARS-CoV-2-specific T-cell and antibody responses within months after infection, the memory T-cell compartment following infection with coronaviruses is reportedly long-lived [7,8,9]. For example, the memory T-cell response to SARS-coronavirus-1—a virus highly related to SARS-CoV-2—is still detectable up to 17 years after infection [7]. Similarly, both memory T- and memory B-cell responses to SARS-CoV-2 appear to be relatively durable following infection [8,9]. Whether such long-term cellular responses are sufficient to protect against severe disease remains to be determined. Nevertheless, in the long term it is the memory T-cell compartment that most likely has the capacity to protect against severe disease caused by different SARS-CoV-2 variants by coordinating both humoral and cellular immunity [10,11,12,13].

Given the reported longevity of cellular immune responses following coronavirus infection and in light of the limited comparison of memory CD4+ T-cell responses following SARS-CoV-2 infection and vaccination, we compared the development of cellular immunity, and in particular long-lived central memory CD4+ T-cells, between the two. We demonstrated that vaccination induces responses that are at least comparable to those induced after infection when measured by either cytokine production at time points beyond the acute peak response or the frequency of SARS-CoV-2-specific central memory CD4+ T-cells.

## 2. Materials and Methods

### 2.1. Cohorts

We compared a number of cohorts before and after vaccination, and after infection. Cohorts were coordinated in accordance with the Helsinki Declaration, with signed consent and relevant ethics approval being obtained, respectively, from patients and the ethics committee at the Ludwig Maximilians Universität München (cohorts 1 and 2: Corona-Register-Study number 20–426, approved 20 July 2021, cohort 3: number 20–735, approved 15 December 2020).

Cohort 1 (C1) comprised hospital staff members: on the day of first vaccination (*n* = 97, 75 female, mean age 40 years from 20 to 69); two weeks after first vaccination (*n* = 41, 31 female, mean age 41 years from 20 to 68); five weeks after first vaccination/two weeks after second vaccination (*n* = 54, 40 female, mean age 43 years from 19 to 68); and three months (10–16 weeks) after first vaccination (*n* = 53, 41 female, mean age 46 years from 22 to 63), of which 49 were measured by activation-induced marker (AIM) assay.

Cohort 2 (C2) comprised patients hospitalized an estimated two-to-four weeks after infection (*n* = 32, 13 female, mean age 64 years from 20 to 88, 30 patients stationary, 11 patients in intensive care).

Cohort 3 (C3) comprised patients recruited by the Department of Vascular Surgery outpatient ward with mild COVID-19 in the majority of cases: three months (6–16 weeks) after infection (*n* = 44, 29 female, mean age 46 years from 20 to 76, 1 patient stationary, no patients in intensive care), of which 13 were measured by AIM assay; six months (5.0–6.9 months) after infection (*n* = 50, 24 female, mean age 43 years from 18 to 74, 1 patient stationary, no patients in intensive care); and twelve months (10–14 months) after infection (*n* = 133, 68 female, mean age 52 years from 18 to 87, 26 patients stationary, 11 patients in intensive care).

Follow-up participation following vaccination was voluntary, for which reason detailed longitudinal studies were not feasible. Any registered participants were included at all of the follow-up time points for which they came.

### 2.2. ELISpot Assays

Ex vivo ELISpot/FLUOROspot assays were performed using the interferon-γ (IFNγ) and interleukin-2 (IL-2) SARS-CoV-iSpot kit from Autoimmun Diagnostika (AID GmbH, Straßberg, Germany). Peripheral blood mononuclear cells (PBMC), drawn into citrate tubes, were isolated on the same or the following day through Ficoll-Paque (GE Healthcare, Munich, Germany), and seeded in duplicate at a density of 2 × 10^5^ cells per well in AIM-V medium. Cells were stimulated for 18 h with the AID SARS-CoV-2 peptide library—containing peptides from the S, N, M, and O proteins—and CD28 antibody, or left unstimulated with CD28 antibody alone as a negative control. As further controls to confirm specific responses to SARS-CoV-2 peptides, we stimulated cells in parallel with peptide libraries cross-reactive with all coronaviruses (PAN), as well as libraries specific to cytomegalovirus, Epstein–Barr virus, and influenza viruses (CEF). Pokeweed Mitogen was used as a positive control. Spot numbers were counted using the AID GmbH iSpot Reader. Samples were excluded if the Pokeweed Mitogen positive control was less than 50 spot-forming units/2 × 10^5^ cells, or if the unstimulated control values were >10 or >20 spot-forming units/2 × 10^5^ cells for IFNγ and IL-2, respectively. Positive responses were stringently defined using the stimulation index (stimulated spot-forming units/2 × 10^5^ cells divided by unstimulated spot-forming units/2 × 10^5^ cells) as clearly positive with a stimulation index of ≥7, and clearly negative with a stimulation index of ≤3. Samples that fell between these values were defined as negative unless the unstimulated control value was >2 spot-forming units/2 × 10^5^ cells and the stimulation index was >3, as described by the manufacturer. All measurements are shown as SI values as opposed to spot-forming units, given the different background levels in the IFNγ and IL-2 assays. SI values were also used to describe the percentage of study participants with positive responses. All ELISpot assays were performed by two laboratory staff members using the same procedure and ELISpot reader settings. SARS-CoV-2-specific IgG antibodies to the spike receptor-binding domain were measured using the Elecsys immunoassay (Roche Diagnostics, Mannheim, Germany) with a positive result indicated by a value > 0.4 U/mL.

### 2.3. AIM Assays

Activation-induced marker (AIM) assays were performed as described by Reiss et al. [14]. The PBMC used in ELISpot assays were plated in round-bottom 96-well plates at a density of 1 × 10^6^ cells/well in AIM-V medium, and incubated with medium alone as an unstimulated control, or with the AID SARS-CoV-2 peptide library—containing peptides from the S, N, M, and O proteins—for 18 h. Cells were blocked with TruStain (BioLegend, Amsterdam, The Netherlands) for 30 min; stained with CD4-Pacific Blue, CD134-FITC, CD25-PE-Dazzle, CCR7-PerCP-Cy5.5, and CD45RO-APC-Cy7 (BioLegend, Amsterdam, The Netherlands) for 60 min at 4 °C; and washed and analyzed on a Cytoflex S cytometer using CytExpert software. Cells were gated on the forward scatter/side scatter live cell gate, and then on the CD3+CD4+ gate for quantification of CD25^hi^CD134^hi^ SARS-CoV-2-specific T-cells and memory T-cell subsets based on expression of CCR7 and CD45RO. All measurements are shown as background subtracted values (CD25^hi^CD134^hi^ stimulated cells minus CD25^hi^CD134^hi^ unstimulated cells as % total CD4+ T-cells). AIM assays were performed using the same flow cytometer with the same settings and CD25+CD134+ gate. Batches of antibodies were the same for all cohorts, with the exception of anti-CCR7, for which reason the CCR7 gate was adjusted relative to the CCR7+ naïve T-cell population.

### 2.4. Statistics

Data were analyzed using GraphPad Prism software using two-tailed Mann–Whitney U-tests with *p* < 0.05 regarded as significant, and one-way analysis of variance with Bonferroni post hoc test.

## 3. Results

### 3.1. Comparison of Cellular Immune Responses Following Infection and Vaccination by ELISpot Assay

**Figure 1:**
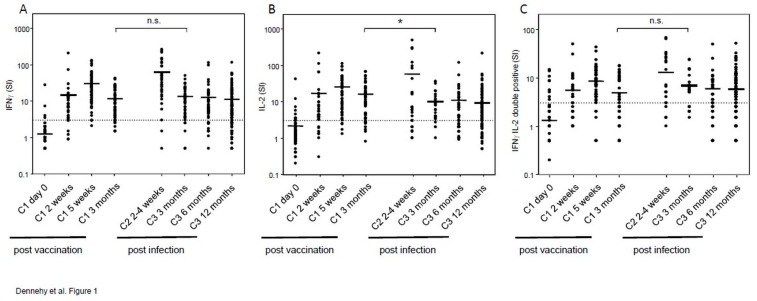
Comparison of cells producing IFNγ (**A**), IL-2 (**B**), or both IFNγ and IL-2 (**C**) measured as stimulation index by ELISpot after stimulation with SARS-CoV-2 antigens at different time points following vaccination (cohort 1) or infection (cohorts 2 and 3). Horizontal bars indicate mean values. The dotted line shows the lower cut-off of SI = 3, below which all results are negative. * indicates significant differences with *p* < 0.05, n.s. indicates non-significant differences.

We initially compared the number of IFNγ-producing cells—as measured by ELISpot after stimulation of PBMC in vitro with a SARS-CoV-2 peptide library—between hospital staff members up to three months after vaccination and patients up to twelve months after infection (Figure 1A). Prior to vaccination, a small proportion of staff members responded to SARS-CoV-2 stimulation, indicative of either previous asymptomatic infection or cross-reactive T-cell responses to seasonal coronaviruses, as previously described (Figure 1A–C) (4% for IFNγ, 6% for IL-2, and 5% for double positive responses) [7]. Consistent with this, only 2% (2 of 93) of hospital staff had measurable antibody responses to SARS-CoV-2 spike receptor-binding domain prior to vaccination. Taken together, these data suggest that the vast majority of cytokine responses are attributable to vaccination de novo, and a minority via boosting of previous asymptomatic infection or pre-existing immunity to seasonal coronaviruses. The IFNγ response was higher at two weeks and reached a maximum at five weeks following vaccination, but decreased substantially at three months post-vaccination.

Levels of IFNγ+ T-cells were remarkably low three months after infection, and declined marginally in a non-significant manner between three and twelve months post-infection (Figure 1A). Although we were not able to measure IFNγ responses in a comparable cohort with mild infection at earlier time points, there was a clearly higher IFNγ response from patients hospitalized with severe infection at 2–4 weeks post-infection. Given the reportedly higher levels of IFNγ+ T-cells in patients with mild compared with severe infection at early time points after infection [15], these data suggest that there is a strong decrease in the IFNγ response after the initial peak, but that the response is comparably stable for three to twelve months after infection, indicative of durable cellular immunity.

Importantly, IFNγ+ T-cell responses three months after vaccination were comparable to those at three, six, and twelve months after infection (mean SI 11.8 ± 1.4, 13.5 ± 1.7, 12.8 ± 3.0, 11.3 ± 1.3, respectively). While the percentage of participants that mounted a T-cell response were also comparable at three months after vaccination and infection (87% and 89%, respectively, for IFNg+ T-cell responses), responses at six and twelve months post-infection were lower at 80% and 75% of participants, respectively. These data indicate that vaccination induces responses that are comparable to those from patients with largely mild infection at three months after infection.

In addition to IFNγ, we measured IL-2 as a second T-helper type 1 cytokine (Figure 1B). While IFNγ is essential for the anti-viral response, IL-2 contributes to both formation of the memory T-cell compartment and maintenance of regulatory T-cells, thereby contributing to an appropriately regulated immune response [16,17]. Surprisingly, levels of IL-2-producing T-cells at three months after vaccination were significantly higher than those produced at three, six, and twelve months after infection (mean SI 15.9 ± 2.3, 8.8 ± 1.6, 10.9 ± 3.0, 9.1 ± 2.1, respectively). Despite this, there were no significant differences between the frequency of T-cells that co-produced IFNγ and IL-2 measured at three months after vaccination with that at three, six, and twelve months after infection (mean SI 4.8 ± 0.7, 5.6 ± 0.9, 5.8 ± 1.2 and 5.8 ± 0.7, respectively). Taken together, these data suggest that vaccination induces responses that are at least comparable to those induced after mild infection when measured at time points well beyond the acute peak response.

### 3.2. Comparison of the Memory CD4+ T-Cell Compartment after Infection and Vaccination by AIM Assay

**Figure 2:**
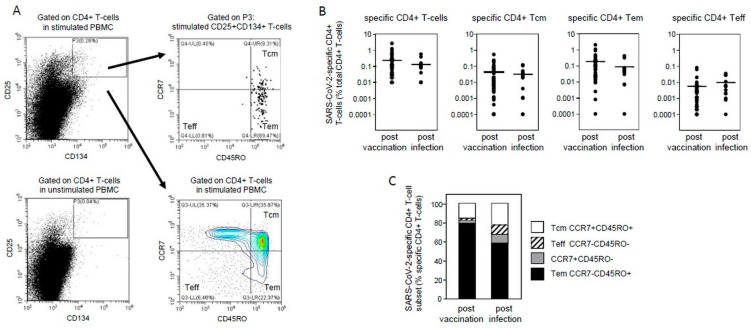
Comparison of SARS-CoV-2-specific memory CD4+ T-cell subsets following vaccination and infection. (**A**) Gating strategy: expression of CD25 and CD134 on CD4+ T-cells from stimulated (above left) or unstimulated (below left) PBMC from a representative vaccinated staff member. The percentages of SARS-CoV-2-specific CD4+ T-cells were calculated by subtracting the values of CD25^hi^CD134^hi^ cells in gate P3 of stimulated PBMC from unstimulated PBMC. The whole CD4+ T-cell gate from stimulated PBMC was used set quadrants on T-cell subsets based on CCR7 and CD45RO expression (below right). Applying these quadrants to cells in the stimulated CD25^hi^ CD134^hi^ gate P3 allowed quantification of SARS-CoV-2-specific memory CD4+ T-cell subsets as percentage of specific CD4+ T-cells (above right). (**B**) Comparison of the frequencies of SARS-CoV-2-specific CD4+ T-cells, specific central memory CD4+ T-cells (Tcm), specific effector memory CD4+ T-cells (Tem), and specific effector T-cells (Teff) as a percentage of total CD4+ T-cells three months after vaccination and infection. Horizontal bars indicate mean values. (**C**) Comparison of the proportions of memory T-cell subsets as percentage of SARS-CoV-2-specific CD4+ T-cells at three months after vaccination and infection.

One caveat of the comparison above is that ELISpot assays preferentially measure immediate responses from effector and effector memory T-cell subsets, and thus may not reflect various T-cell populations differentially induced by infection versus vaccination [5]. We therefore directly compared the frequency of SARS-CoV-2-specific memory CD4+ T-cell subsets at three months after infection and vaccination. For this purpose we used AIM (activation-induced marker) assays, based on co-expression of CD25 and CD134 following in vitro stimulation, which reveal a broad range of specific T-cells including CCR7+CD45RO+ central memory T-cells (Tcm), CCR7-CD45RO+ effector memory T-cells (Tem), and CCR7-CD45RO- effector T-cells (Teff) (Figure 2A) [14]. Randomly sampled participants demonstrated no significant differences in the levels of cytokine-producing T-cells measured by ELISpot compared with their parental cohorts, indicating that they are representative of their respective cohorts (sampled IFNγ SI 9.4 ± 1.1 versus parental 11.8 ± 1.4 after vaccination, and sampled IFNγ SI 14.6 ± 3.0 versus parental 13.5 ± 1.7 after infection). We found that marginally more SARS-CoV-2-specific CD4+ T-cells were induced three months after vaccination than after infection, although these differences were not significant (Figure 2B) (mean 0.245% ± 0.063 versus 0.136% ± 0.045). Similarly, the frequencies of specific central memory CD4+ T-cells and the proportions of central memory T-cells as a percentage of specific CD4+ T-cells were comparable after vaccination and infection (Figure 2B,C) (mean 0.045% ± 0.013 versus 0.033% ± 0.011 and mean 16% ± 2 versus 23% ± 6, respectively). Although the frequencies of specific effector memory CD4+ T-cells and effector CD4+ T-cells after vaccination were approximately double and half, respectively, of those after infection, these differences were not significant (Figure 2B) (mean Tem 0.189% ± 0.049 versus 0.090% ± 0.035 and mean Teff 0.006% ± 0.002 versus 0.010% ± 0.003, respectively). Nevertheless, the proportions of effector memory T-cells as a percentage of specific CD4+ T-cells were significantly higher (Figure 2C) (mean 79% ± 2 versus 58% ± 6), whereas proportions of specific effector CD4+ T-cells were significantly lower following vaccination compared with infection (Figure 2C) (mean 2% ± 1 versus 10% ± 3). These data suggest that SARS-CoV-2-specific central memory CD4+ T-cells, indicative of long-term memory, are comparably induced following vaccination and infection, despite the differential induction of effector and effector memory CD4+ T-cell subsets.

## 4. Discussion

During the course of the SARS-CoV-2 pandemic we measured immune responses from infected patients and vaccinated hospital staff members from a number of cohorts. Here, we collate these data to retrospectively compare the development of cellular immune responses, and particularly the central memory CD4+ T-cell compartment required for development of long-term immunity.

We show that IFNγ+ T-cell responses after infection are remarkably stable between three and twelve months post-infection (Figure 1A). In longitudinal studies, we and others have previously shown significant decreases in the IFNγ response up to twelve months after infection [8,9,18]. The results shown here nevertheless underline the marginal decline of such responses, particularly a biphasic decline with a strong decrease in the first three months, followed by a comparably stable second phase indicative of durable cellular immunity. A similar biphasic decline with a strong decrease in, approximately, the first three months post-infection was previously described [19].

Levels of IFNγ+ T-cells at three months after vaccination were comparable with those at three, six, and twelve months after infection (Figure 1A). By contrast, IL-2+ T-cells at three months after vaccination were significantly higher than at any time point after infection (Figure 1B). Nevertheless, there were no significant differences in the frequencies of T-cells producing both IFNγ and IL-2 at three months post-vaccination and three, six, and twelve months post-infection (Figure 1C). From adoptive transfer and vaccination studies it is known that the quality of immune responses—measured in part by the frequency of T-cells that produce multiple cytokines required for anti-viral but also regulatory responses—is key to controlling viral infection [17,20,21]. The results presented here suggest that the quality of cellular responses after vaccination is at least comparable to that induced after infection. Higher IL-2 responses following vaccination may be due to shorter exposure to vaccine antigen compared with persistent antigen after infection, and consequent enhanced development of uncommitted IL-2+IFNγ- memory CD4+ T-cells [22,23], although this remains to be tested. Increased IL-2 responses may also indicate a general enhanced development of memory T-cells following vaccination given the role of IL-2 in formation of the memory T-cell compartment [17], for which reason we quantified memory T-cells and their subsets after vaccination and infection using AIM assays.

The frequencies of specific CD4+ T-cells were comparable at three months after vaccination and infection, and the frequencies and also proportions of specific central memory CD4+ T-cells as a percentage of specific CD4+ T-cells were similarly comparable (Figure 2B,C). A recent study found comparable levels of SARS-CoV-2-specific central memory CD4+ T-cells one month after vaccination compared with those from patients that had recovered from a previous infection [4], which supports the results we present here. In contrast to central memory T-cells, the proportions of effector memory T-cells as a percentage of specific CD4+ T-cells were significantly lower, and the proportion of effector T-cells significantly were higher after infection than after vaccination (Figure 2C). A likely explanation may be that robust viral infection forces higher levels of differentiation of effector memory T-cells into effector T-cells than that following shorter-term exposure to vaccine antigen. Using our routine staining panel we were not able to more precisely define whether these CCR7-CD45RO- effector T-cells are terminally differentiated CD45RA+ T-cells. Given the longevity of central memory T-cells, together with the relative stability of T-cell responses beyond the peak of response after infection indicated by ELISpot assays, our data suggest that the long-lived memory T-cell compartment is likely to be similar after vaccination and infection.

Our study has some limitations. Firstly, CCR7 expression is not a stable marker of central memory T-cells since it is downregulated after stimulation. However, the vast majority of central and effector memory T-cells retain their CCR7 expression status in the first 24 h after stimulation, thereby allowing an estimation of central memory T-cell frequency as previously described [8,24]. Secondly, the only way in which we could measure the earliest responses after infection was from hospitalized patients with severe disease, which are not intended to be directly compared with vaccinated staff members or patients with mild infection. Rather, the levels of IFNγ+ T-cells, which are reportedly lower in patients with severe than mild infection [15], are presented to put the surprisingly low IFNγ+ T-cell responses from three months into context. We previously demonstrated that the duration of acute phase symptoms correlates with levels of cellular immunity [18]. Given that most of the patients in cohort 3 described here had mild COVID-19, our comparison is limited to patients with mild COVID-19 compared with persons vaccinated with BioNTech/Pfizer BNT162b2. We compared memory CD4+ T-cell subsets at only one time point after infection and vaccination. The nonsignificant differences between the proportions of central memory CD4+ T-cells may well become significant at later time points; for example, as a result of contraction of the short-lived effector T-cell subset that is more prominent after infection than vaccination. The comparable levels of central memory CD4+ T-cells that we present here at three months post-infection/vaccination may not necessarily protect equally well against the development of severe disease following reinfection or break-through infection. This is reportedly also not the case, at least for the SARS CoV-2 delta variant where there is a greater risk of break-through/reinfection and severe disease after vaccination than after infection [25]. Major reasons for this difference are likely the greater breadth of the T-cell response to multiple antigens after infection, which correlates with the development of mild as opposed to severe disease [26], as well as the development of resident memory T-cells after infection. Nevertheless, our data demonstrate, somewhat surprisingly, that levels of SARS-CoV-2-specific CD4+ T-cells and central memory CD4+ T-cells are comparable at three months following vaccination and infection. Lastly, we did not measure resident memory T-cells, which are induced following infection but not by current vaccination strategies, but instead limited our study to T-cells that are measurable in the periphery.

## 5. Conclusions

Taken together, our results demonstrate that SARS-CoV-2-specific cellular immunity—determined by cytokine production at time points beyond the acute peak response as well as the frequency of central memory CD4+ T-cells indicative of long-term memory—are comparably induced following vaccination and infection.

## Data Availability

The data presented in this paper are available on request from the corresponding author.
